# Mechanism of Nrf2/miR338-3p/TRAP-1 pathway involved in hyperactivation of synovial fibroblasts in patients with osteoarthritis^☆^

**DOI:** 10.1016/j.heliyon.2023.e21412

**Published:** 2023-10-21

**Authors:** Peng Jie, Ya Wu, Changzhi Song, Yi Cheng, Yunfei Liu, Kang Chen

**Affiliations:** Department of Orthopaedics, the Fourth Affiliated Hospital of Nantong University (The First people's Hospital of Yancheng), Yancheng 224006, China

**Keywords:** Osteoarthritis, TRAP-1, Nrf2, miR338-3p, Synovial fibroblasts, Oltipraz

## Abstract

Our previous study has confirmed that miR338-3p/TRAP-1 axis was involved in regulation of hyperactivation in human synovial fibroblasts (HFLS) of patients with osteoarthritis (OA). Here, we aim to further investigate the underlying causes of the abnormal activation miR338-3p/TRAP-1 at the molecular level. Our results showed that the decrease of NF-E2-related factor 2(Nrf2) was the direct cause of downregulation of miR338-3p and upregulation of TRAP-1 protein expression in HFLS of OA patients. Furthermore, we also found that the phosphorylation and nuclear entry of Nrf2 protein were significantly reduced in HFLS of OA patients than that of normal individuals, and both of them were positively correlated with miR338-3p levels. Bioinformatics analysis, luciferase assay, and CHIP experiment together indicated that Nrf2 could positively regulate the transcription of miR338-3p by binding to the Transcription Factor Binding Sites (TFBS) on its promoter. It was confirmed by in vitro assays that oltipraz (agonists of Nrf2) treatment effectively inhibited the hyperactivation of HFLS induced by TGF-β1, and the effects of oltipraz could be reversed by the exogenous TRAP-1. In short, our research has revealed for the first time that Nrf2/miR338-3p/TRAP-1 pathway was involved in hyperactivation of HFLS in OA patients, Nrf2 has the potential to be used as therapy and new drug target of OA.

## 1 Introduction

Osteoarthritis (OA) is a common degenerative joint disease in the elderly. Recent studies have shown that OA is characterized by various pathological changes, including synovial inflammation, articular cartilage injury, bone remodeling and so on [1]. Synovitis is considered to play an important role in aggravating the progression of OA [2]. It is conformed that hyperactivation of human synovial fibroblasts (HFLS) could exacerbate the synovitis in OA patients via secreting the inflammatory factors such as tumor necrosis factor α (TNF-α), interleukin-1β (IL-1β) and interleukin-6(IL-6) [3]. Furthermore, the hyperactivated FLS can recruit monocytes and lymphocytes to the synovium by expressing adhesion molecules such as intercellular adhesion molecule1 (ICAM-1) and vascular cell adhesion molecule1 (VCAM-1), and further aggravate the formation of inflammatory microenvironment [4]. Clinical studies suggested that the synovial fibrosis and hyperplasia caused by hyperactivated synovial fibroblasts were common in patients with advanced OA. Currently, there are no specific drugs of joint stiffness caused by synovial fibrosis and hyperplasia in OA patients.

Our previous study demonstrated that TNF receptor associated protein 1 (TRAP-1) was highly expressed and caused the hyperactivation of HFLS in synovial tissues of OA patients, and the upregulation TRAP-1 is attributed to the decrease of miR338-3p. We also confirmed that the overexpression of exogenous miR338-3p could effectively suppress the hyperactivation of HFLS by inhibiting protein expression of TRAP-1 [5]. However, the reason for the downregulation of miR338-3p in the HFLS of OA patients still needs further exploration. In this study, our primary goal is to further investigate the underlying causes of the abnormal activation miR338-3p/TRAP-1 pathway in OA patients at the molecular level, expecting to provide new insights for the mechanism research of OA and provide new targets for the development of new drugs for OA treatment.

## 2 Materials and methods

### 2.1 Collection of synovium tissues

The synovium tissues were obtained from of OA patients and normals with mechanical injuries from the Orthopedics department of Yancheng City No.1 People's Hospital in from June 1st, 2021 to December 1st, 2021. Informed consents were obtained from all the patients in accordance with the standards set by the Ethics Committee of the First people's Hospital of Yancheng. All protocols concerning the use of human samples were approved by the Ethics Committee (Approval No.2021-K-119).

### 2.2 Cell culture

Synovium tissues obtained from surgery were rapidly used for preparation of the HFLS by using the reported method [5,6]. The adherent cells were incubated in Dulbecco's Modified Eagle Medium (DMEM, Thermofisher, CA, USA) supplemented with 10 % fetal bovine serum (FBS, Thermofisher) in an incubator (MCO-20AIC, SANYO, Japan) with 5 % CO2 at 37 °C. The cells were passaged using 0.25 % trypsin when they reached about 70 % confluency. Only cells of passage 2 were included in this study.

### 2.3 Measurements of miR338-3p, phosphorylated Nrf2 protein and its nuclear aggregation in HFLS, and pairs correlation analysis between them

Bioinformatics analysis showed that there was likely to be an upstream and downstream relationship between the nuclear transcription factor Nrf2 is and miR338-3p. Therefore, we analyzed the expression correlation between them in HFLS from OA patients and normal control subjects. About 1 × 10^7^ HFLS from each patient and normal control subject were used to extract total RNA which were then used for the measurement of miR338-3p levels using real-time quantitative PCR (RT-qPCR). At the same time, about 1 × 10^7^ HFLS were also used for proteins extraction which were used for detecting the phosphorylated Nrf2 protein by using western blotting. Furthermore, these cells were also used for nucleus proteins extraction by NE-PER Nuclear and Cytoplasmic Extraction Reagent (78833, Thermofisher) which were used for detecting the nucleus entry of Nrf2 protein. Spearman's method was used for correlation analysis by SPSS 20.0 software (IBM Corp., NY, USA).

### 2.4 Identification of the binding site of miR338-3p on the 3′UTR of TRAP-1 mRNA

The experimental design, implementation and data analysis were generally consistent with that of our previous studies [5]. Briefly, 3′UTR of TRAP-1 mRNA was extracted and used to construct pGL3-wt-TARP-1 at downstream of luciferase gene. At the same time, we also prepared pGL3-wt-TARP-1 which contains a misspelled miR338-3p predicted binding site through point mutation. After that, the reporter vector pGL3-wt-TARP-1 and miR338-3p mimics or inhibitor were co-transfected into 293 cells respectively. At 48h after transfection, the luciferase assays were performed using a Dual Luciferase Reporter Assay kit (E1910, Promega Corporation, WI, USA), and the changes of intracellular luciferase activity were used to evaluate whether miR338-3p could bind to TRAP-1 mRNA 3′UTR.

### 2.5 Verification of the Transcription Factor Binding Sites (TFBS) of Nrf2 in miR338-3p promoter

Bioinformatics analysis suggested the presence Nrf2 TFBS in the miR338-3p promoter, and we also confirm that Nrf2 protein activity (phosphorylation and aggregation) maintains a positive correlation with miR338-3p expression in HFLS from OA patients and normal controls. To this end, we performed the validation experiments on the TFBS. The coding sequence (CDS) of human Nrf2 (NM_006164.5) was amplified by using human complementary DNA (cDNA) as a template with the primers 5′-GGAATTCATGATGGACTTGGAGCTGC-3′ and 5′-CGGGATCC CTAGTTTTTCTTAACATCTGG-3’. The PCR products were digested and used to construct the expression vector pcDH-Nrf2. Then we searched for the location of the precursor of miR338-3p (pri-miR338) in human genome and selected a 2.0 kb DNA sequence upstream of the transcription start site as the promoter region. Then, we predicted the promoter sequence of miR338-3p by using “Promoter 2.0” software (http://www.cbs.dtu.dk/services/Promoter), which were then cloned to construct pcDNA-pro (miR338-3p)-GFP at upstream of GFP gene. We then transfected the pcDNA-pro (miR338-3p)-GFP into 293T cells. At 48 h after transfection, the promoter activity was evaluated by GFP expression. A vector pcDNA-pro (−/−)-GFP with a universal CMV promoter removed was used as a negative control (NC).

Then we predicted TFBS of Nrf2 in miR338-3p promoter by using “JASPAR” (http://genexplain.com/transfac). The promoter of miR338-3p (253bp) was amplified by PCR using human genomic DNA as the template. The PCR primers, 5′-GGGGTACCCTTCTGTTACCAAACTGTGGGTGA-3′ (forward) and 5′-CCCAAGCTTGGGAAGAAGGTCGTACAGGGGGGCC-3′ (reverse). The PCR product was digested and used for the construction of pGL3-wt (TFBS)-miR338-3p which carried a wild-type TFBS of Nrf2. Then, the TFBS in the pGL3-wt (TFBS)-miR338-3p was mutated from 5′-GCTCCGTCA-3′ to 5′-CAGCTCTCG-3′ to construct the pGL3-mt (TFBS)-miR338-3p which carried a mutated TFBS. Then the vectors were transfected in 293 cells according with the instructions of Lipofectmaine 2000 (16680191, Thermofisher). The experimental groups: control group (without transfection), pGL3-wt (TFBS)-miR338-3p transfection group, pGL3-mt (TFBS)-miR338-3p transfection group, pGL3-wt (TFBS)-miR338-3p + pcDH-Nrf2 co-transfection group, pGL3-mt (TFBS)-miR338-3p + pcDH-Nrf2 co-transfection group and pGL3-wt (TFBS)-miR338-3p + pcDH vector co-transfection group. At 48h after transfection, the luciferase assays were performed and the intracellular luciferase changes would be used to evaluate whether Nrf2 could regulate the gene transcription guided by the miR338-3p promoter by binding to the TFBS.

### *2.6* *Chromatin immunoprecipitation-PCR (*ChIP-PCR*)*

The HFLS transfected with pcDH-Nrf2 for 72 h were harvested and subjected to ChIP-PCR using the EZ ChIP Kit (17–371, Millipore, MI, USA) according to the manufacturer's instructions. The primers used for Reverse Transcription-Polymerase Chain Reaction (RT-PCR) were 5′-TCTGCTTCTCTACGCATGACA-3’ (forward) and 5′-CGTCCCACGACCGACTGAC-3’ (reverse). The theoretical PCR product of 68 bp carrying the predicted TFBS of Nrf2 was analyzed by 2 % agarose gel electrophoresis. Four micrograms of Nrf2 primary antibody (ab137550, Abcam, Cambridge, USA) was used for purification of the target protein.

### 2.7 Effects of oltipraz treatment on the Nrf2/miR338-3p/TRAP-1 pathway in HFLS induced by TGF-β1

The preparation of recombinant lentivirus Lv-TRAP-1 and its infection schemes to HFLS referred to our previous reports [5]. HFLS in the logarithmic growth phase were inoculated into 6-well culture plate at 1 × 105 cells per well. After overnight culture, the cells were randomly divided into 5 groups: control group (without treatment), TGF-β1 induction group, solvent + TGF-β1 induction group, oltipraz pretreatment + TGF-β1 induction group and oltipraz pretreatment + Lv-TRAP-1+TGF-β1 induction group. Lv-TRAP-1 was added 48 h before cell inoculation, the final concentration of TGF-β1 (ab50036, Abcam) was 10 ng/ml and the induction time was set 72 h. The oltipraz (ab141957, abcam.10 mM mother liquor prepared with DMSO) was added to the cell culture with the final concentration of 10 μM at 2h before the addition of TGF-β1, and the methyl sulfoxide (DMSO, Sigma-Aldrich, MO, USA) with the same volume as oltipraz was used as solvent control [5,7]. At the endpoint, total RNA and proteins were isolated from the cells of each group and subjected to RT-qPCR for detection of miR338-3p expression levels and western blotting for detection the expression of TRAP-1 and fibroblast activation proteins FAP, α-SMA and Collagen I. Furthermore, we also isolated the nuclear proteins of HFLS which were used for detection of nucleus entry of Nrf2 protein by western blotting.

### 2.8 Validation for upregulation of TRAP-1 expression depends on the Nrf2/miR338-3p/TRAP-1 pathway in HFLS

Since oltipraz is a non-specific agonist of Nrf2, we believe it is necessary to verify whether the upregulation of TRAP-1 expression by oltipraz depends on Nrf2/miR338-3p/TRAP-1 pathway. Our strategy is to observe whether the targeted silencing of Nrf2 affects the upregulation of TRAP-1 by oltipraz in HFLS. Firstly, we designed and selected an siRNA (5′-GAGCTAGATAGTGCCCCTG-3′) targeting human Nrf2 and constructed the shRNA expression vector pshRNA-Nrf2, which was further used to produce the recombinant lentivirus Lv-shRNA-Nrf2 according to our previous method [5]. Next, we inoculated HFLS infected by lentivirus for 72 h into a 6-well culture plate with a density of 1 × 10^5^ cells per well and cultured for 24 h, followed by a final concentration of 10 μM oltipraz was added to cell culture medium. At 24 h after incubation, cells were collected and used to extract total RNA which were used to detect miR338-3p levels by RT-qPCR, and total protein was extracted for TRAP-1 protein expression detection using western blotting.

### 2.9 Enzyme linked immunosorbent assay (ELISA)

The experimental treatments and groupings were the same as those used in 2.7, the HFLS in the logarithmic growth phase were seeded in 96-well culture plate at 5 × 10^4^ cells per well. After 72 h of TGF-β1 induction, the supernatants were collected and centrifuged at 10,000×*g* and 4 °C for 2 min, and then were used to detect the inflammatory factors TNF-α, IL-1β and IL-6 following the instructions of ELISA kits (ES24RB, EH2IL6 and BMS224-2, Thermofisher) respectively.

### 2.10 Cell proliferation assay

The experimental treatments and groupings were the same as those used in 2.7, the HFLS in the logarithmic growth phase were seeded in 96-well culture plate at 5 × 10^4^ cells per well and inducted under normal condition for another 72h, and the proliferation activity were detected by CCK-8 method at 24, 48, and 72 h after cultivation. Briefly, 10 μl CCK-8 solution (CK-04, Dojindo, Japan) was added into the culture medium, and the cells were transferred to incubator and cultured under normal conditions for another 2h before measurement of absorbance at 450 nm.

### 2.11 RNA isolation and RT-qPCR

In this study, RT-qPCR was mainly used to detect the relative contents of miR338-3p in sample of cells. Briefly, after 2 rounds of Dulbecco's Phosphate-Buffered Saline (dPBS) cleaning, approximately 1 × 10^6^ cells were fully lysed by 1 ml Trizol Reagent (15596018, Thermofisher), followed by extracting total RNA using GenElute mRNA Miniprep Kit (RTN350. Sigma-Aldrich), and finally measuring its concentration using a UV fluorescence photometer (UV-1700, Shimadzu, Japan). Two micrograms RNA of each sample was used to produce cDNA with the 3′-specific primers, 5′-TACCTTGCGAAGTGCTTAAAC-3′ for human U6 snRNA and 5′-GTCGTATCC AGTGCGTGTCGTGGAGTCGGCAATTGCACTGGATACGAACAACAA-3′ for miR338-3p. After that, quantitative-PCR was carried out using a GoTaq qPCR Master Mix (Promega Corporation) and the TP800 System (Takara Bio Inc.) with three repeated reactions. The differences between groups in expression levels of miR338-3p were analyzed using the 2^−ΔCt^ method, and U6 snRNA was used as internal reference. The pairs of PCR primers were as follows: U6-forward 5′-GTGCTCGCTTCGGCAGCACAT-3′ and U6-reverse 5′-TACCTTGCGAAGTGCTTAAAC-3′; miR338-3p-forward 5′-GCCGGCGCCCGAGCTCTGGCTC-3′ and miR338-3p-reverse 5′-TCCAGCATCAGTGATTTTGTTG-3′.

### 2.12 Western blotting

Ten micrograms of proteins of each group were separated by 11 % SDS-PAGE and transferred onto nitrocellulose membranes. The blots were incubated with primary antibodies against human Nrf2(ab137550, Abcam. 1:400), TRAP-1 (ab2721, Abcam. 1:700), α-SMA (ab108424, Abcam. 1:500), FAP (ab227703, Abcam. 1:600), Collagen I (ab34710, Abcam.1:400), HDAC1 (ab7208, Abcam. 1:500), *p*-Nrf2 (ab76026, Abcam. 1:300) and β-actin (ab6276, Abcam. 1:2000) overnight at 4 °C, following incubated with the corresponding HRP-conjugated secondary antibody (ab205718, Abcam.1:3500). After washing the membranes twice with Tris Buffered Saline with Tween (TBST, pH = 7.4) and the bands in the membranes were visualized by enhanced chemiluminescence (ECL, Pierce, USA). After incubation for 2 min, the membranes were exposed to x-ray films. The protein bands were scanned and analyzed. The levels of these proteins were calculated as the ratio of the intensity of the specified protein to that of β-actin by using ImageJ2x software (National Institutes of Health, Maryland, USA). The intranuclear Nrf2 protein levels were calculated as the optical density ratio of intranuclear Nrf2 to HDAC1, and the phosphorylated Nrf2 was calculated as optical density ratio of phosphorylated Nrf2 to its total.

### 2.13 Statistical analysis

The data are shown as the means ± standard deviation (SD) of three independent experiments. All statistical data were analyzed using SPSS GradPack version 20.0 statistical software and GraphPad Prism 7.0 (GraphPad Software, Inc., La Jolla, CA, USA). Comparisons between groups were performed using a two-tailed Student's *t*-test or one-way ANOVA with a post-hoc Tukey test. Differences were considered statistically significant when *p* < 0.05.

## 3 Results

### *3.1* *T*he expression levels of miR338-3p were significantly decreased, while *phosphorylated protein expression and nuclear aggregation of Nrf2 were significantly enhanced in HFLS of OA patients than that of normals*

**Fig. 1:**
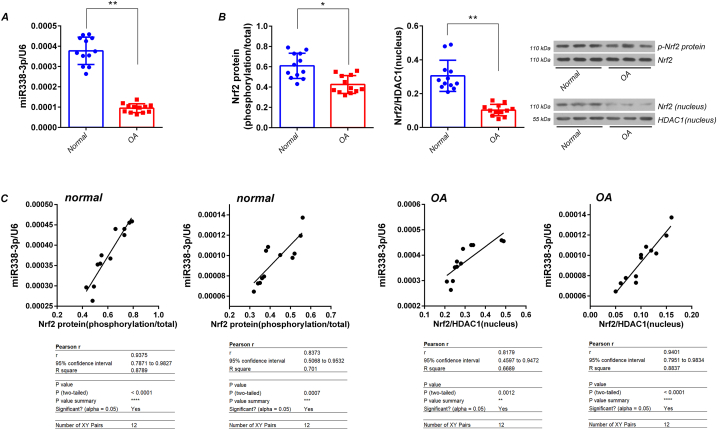
Detection of expression of miR338-3p and phosphorylation and nuclear entry of Nrf2 protein in HFLS of patients with OA and normal individuals A)RT-qPCR for detection of miR338-3p levels in HFLS. B)Detection of the phosphorylation and intranuclear aggregation of Nrf2 protein in HFLS using western blotting. C)Correlation analysis. The data are expressed as the mean ± standard deviation (SD). **p < 0.01, *p < 0.05 (*t*-test). Twelve cases in each group (n = 12).

The results of RT-qPCR showed that the expression levels of miR338-3p were significantly decreased in HFLS of OA patients than that of normal individuals (*p* < 0.01, *vs.* normals) (Fig. 1-A). Western blotting showed that the phosphorylation and nuclear aggregation of Nrf2 protein were significantly decreased in HFLS of OA patients compared to those of normal individuals (*p* < 0.05, *vs.* normals) (Fig. 1-B). Comprehensive analysis showed that phosphorylation and nuclear aggregation of Nrf2 protein were positively correlated with miR338-3p levels in HFLS of both OA patients and normal individuals (Fig. 1-C).

### 3.2 MiR338-3p can bind to 3′-UTR of TRAP-1 mRNA

**Fig. 2:**
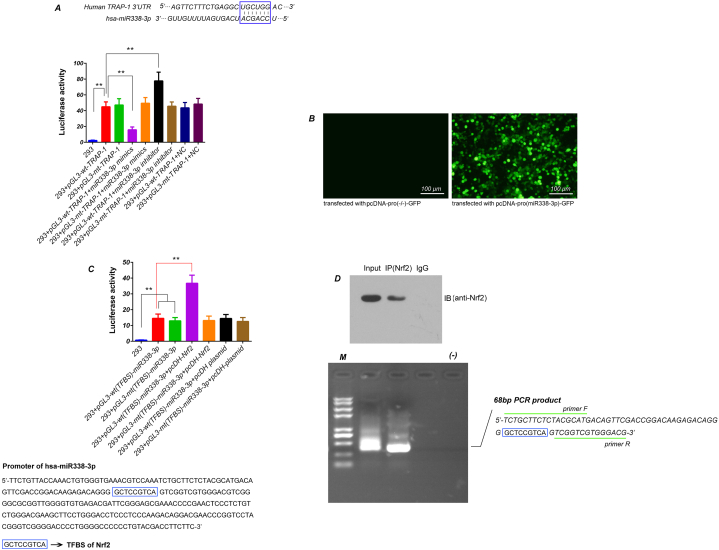
Luciferase assay and ChIP-PCR A) Luciferase method was used to verify binding sites of miR338-3p in TRAP-1 mRNA. Upper, bioinformatics prediction of the binding sites of miR338-3p seed region in 3′-UTR of TRAP-1 mRNA. Down, analysis of the effect of miR338-3p mimics, inhibitor and NC on the luciferase activity in 293 cells after 48h of co-transfection. B) Evaluation of miR338-3p promoter activity via observing protein GFP. C) Verification of the TFBS of Nrf2 in the miR338-3p promoter. Upper, bioinformatics prediction of TFBS. Down, differences analysis of luciferase activity in 293 cells after 48h of co-transfection. The intracellular luciferase activity is expressed as the ratio of firefly luciferase to renilla luciferase. D)Chip-PCR. Upper, immunoprecipitation of Nrf2 protein in HFLS. Down, gel electrophoresis of the Nrf2 binding DNA fragments containing TFBS in the miR338-3p promoter amplificated by using eluents of immunoprecipitated Nrf2 as a template. M, DL2,000 DNA Marker. (−) Negative control (without template). **p < 0.01, *p < 0.05. The tests were carried out on three biological triplicates, and data are expressed as the mean ± SD.

Bioinformatics analysis suggested there was a 7-base binding sites 5′-UGCUGGA-3′ of miR338-3p seeds region in TRAP-1 mRNA 3′UTR (Fig. 2-A). Luciferase results showed that there was the strong luciferase activity detected in 293 cells transfected with pGL3-wt-TRAP-1 or pGL3-mt-TRAP-1 (p＜0.01,vs. cell group). The miR338-3p mimics significantly decreased the luciferase activity in 293 cells transfected with pGL3-wt-TRAP-1 (*p* < 0.01, vs. 293 cells transfected with pGL3-wt-TRAP-1 group), while the miR338-3p inhibitor significantly increased luciferase activity in these cells (*p* < 0.01, vs. 293 cells transfected with pGL3-wt-TRAP-1 group). However, both miR338-3p mimics and miR338-3p inhibitor did not affect the luciferase activity in 293 cells transfected with the pGL3-mt-TRAP-1 (*p* > 0.05, vs. 293 cells transfected with pGL3-mt-TRAP-1 group). Furthermore, miR338-3p NC did not affect the luciferase activity in both pGL3-wt-TRAP-1 and pGL3-mt-TRAP-1 transfected 293 cells (*p* > 0.05) (Fig. 2-B). The above results collectively indicated that miR338-3p could suppress TRAP-1 protein expression by binding to 3 'UTR of its mRNA.

### 3.3 Nrf2 can regulate the transcription of miR338-3p by binding to its promoter

We obtained a promoter of miR338-3p (253 bp) through bioinformatics prediction (Fig. 2-C). At 48 h after transfection, the strong GFP expression could be observed in most 293 cells transfected with pcDNA-pro (miR388-3p)-GFP and there was no GFP could be observed not in pcDNA-pro (−/−)-GFP transfected cells, indicating that the miR338-3p promoter has the ability in guiding downstream gene transcription (Fig. 2-B). Furtherly, based on the bioinformatics analysis, we also found a conserved TFBS 5′-GCTCCGTCA-3′ of Nrf2 in miR388-3p promoter (Fig. 2-C). Luciferase assay showed that overexpression of Nrf2 significantly increased the luciferase activity in 293 cells transfected with pGL3-wt (TFBS)-miR338-3p (*p* < 0.01, vs. 293 cells transfected with pGL3-wt (TFBS)-miR338-3p group), but had no effect in 293 cells transfected with pGL3-mt (TFBS)-miR338-3p (*p* > 0.05, vs. 293 cells transfected with pGL3-mt (TFBS)-miR338-3p group) (Fig. 2-C). ChIP-PCR further validated the existence of TFBS of Nrf2 in miR388-3p promoter once again. The results of gel electrophoresis showed that the short DNA fragments belonging to miR338-3p promoter and containing TFBS of Nrf2 was successfully identified by PCR in eluents of immunoprecipitated Nrf2 protein in HFLS (Fig. 2-D).

### 3.4 Oltipraz treatment effectively inhibited the TGF-β1 induced TRAP-1 expression in HFLS via Nrf2/miR338-3p/TRAP-1 pathway

**Fig. 3:**
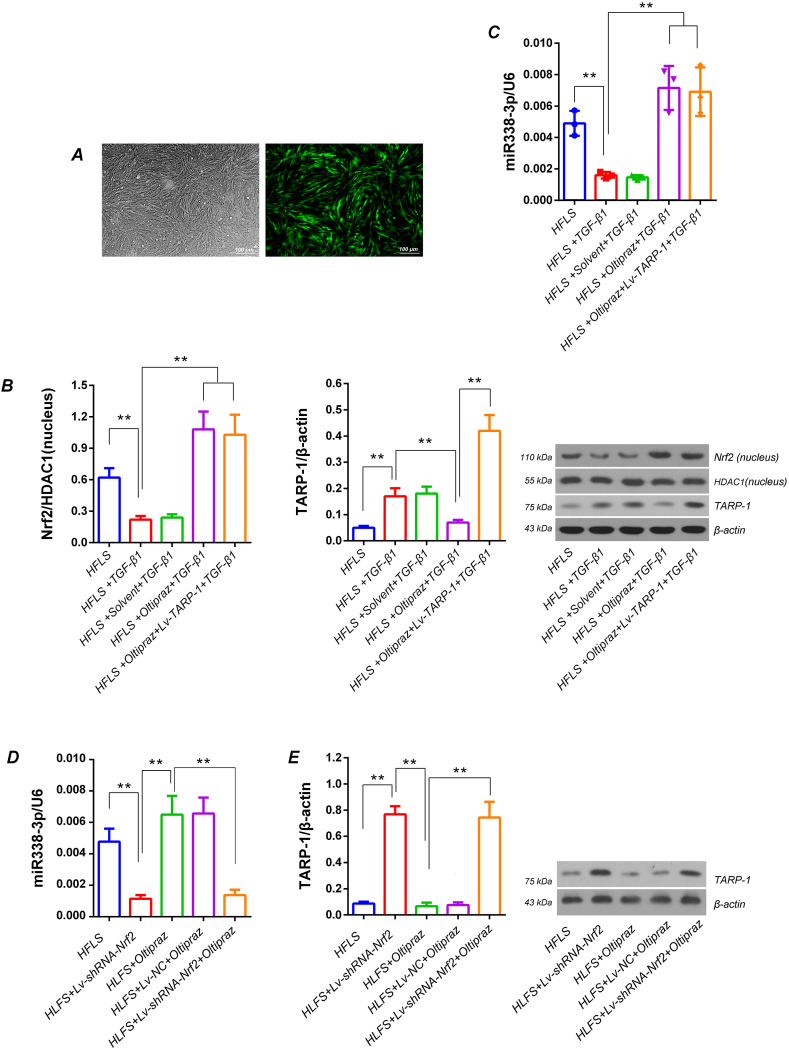
The effect of oltipraz pretreatment on intranuclear aggregation of Nrf2 and expression of TRAP-1 and miR338-3p in HFLS induced by TGF-β1 A) Evaluating the efficiency of lentivirus infection on HFLS through GFP expression. After 72 h of infection, the viral infection efficiency is expressed as the ratio of GFP positive cells to the total number of cells. Five views were randomly selected for statistical analysis. B) The intranuclear aggregation of Nrf2 and TRAP-1 protein expression were detected by western blotting. C, D) Determination of miR338-3p levels in HFLS by RT-qPCR. E) Western blotting for detection of TRAP-1 protein expression in HFLS. The tests were carried out on three biological replicates, and the data are expressed as the mean ± SD. **p < 0.01, *p < 0.05.

At 72 h after infection, the GFP positive cells counting results suggested that the infection efficiency of lentivirus on HFLS was not less than 95 % (Fig. 3-A). Western blotting results indicated that the TRAP-1 protein expression was significantly increased in the TGF-β1 induced HFLS (p＜0.01, vs. cell group). Oltipraz pretreatment could significantly weaken effects of TGF-β1 on TRAP-1 protein in HFLS (*p*＜0.01, vs. TGF-β1-induction group), while the effects of oltipraz was significantly reversed by Lv-TRAP-1 infection (*p*＜0.01, vs. oltipraz pretreatment + TGF-β1 induction group).The protein detection results also showed that oltipraz pretreatment could effectively reverse the TGF-β1 caused decrease of Nrf2 protein intranuclear aggregation in HFLS (p＜0.01, vs. cell group; *p*＜0.01, vs. TGF-β1 induction group). Moreover, the effects of oltipraz were blocked by Lv-TRAP-1 infection in HFLS (*p*＜0.01, vs oltipraz pretreatment + TGF-β1 induction group) (Fig. 3-B). RT-qPCR data showed that the expression levels of miR338-3p were significantly decreased in TGF-β1-induced HFLS (p＜0.01, vs. cell group), the oltipraz pretreatment could significantly upregulate the expression of miR338-3p in TGF-β1 induced HFLS (*p*＜0.01, vs TGF-β1 induction group). However, the effects of oltipraz on miR338-3p was not affected by Lv-TRAP-1 in TGF-β1 induced HFLS (*p*＞0.05, vs. oltipraz pretreatment + TGF-β1-induction group)(Fig. 3-C). All these data not only clarified the change rules of Nrf2, miR338-3p and TRAP-1 in the HFLS, but also investigated that the relationship between them should be Nrf2/miR338-3p/TRAP-1.

### 3.5 Nrf2 gene silencing can reverse the effects of oltipraz on the upregulation of miR338-3p and downregulation of TRAP-1 protein in HFLS

The RT-qPCR results showed that compared with the HFLS group, the miR338-3p expression was significantly decreased in HFLS infected with Lv-shRNA-Nrf2 (*p*＜0.01,*vs.* HFLS group). The oltipraz pretreatment significantly upregulated the expression levels of miR338-3p in HFLS (*p*＜0.05,*vs.*HFLS group), and the effects of oltipraz on miR338-3p was reversed by Lv-shRNA-Nrf2 infection in HFLS (*p*＜0.01,*vs.*HFLS + oltipraz group) (Fig. 3-D). The western blotting showed that the changes of TRAP-1 protein expression in HFLS of each treatment group was completely opposite with miR338-3p levels (Fig. 3-E). These results collectively indicated that oltipraz pretreatment could upregulate miR338-3p and downregulate TRAP-1 protein expression by targeting activation on Nrf2 protein in HFLS.

### 3.6 Oltipraz treatment suppressed the hyperactivation in HFLS induced by TGF-β1

**Fig. 4:**
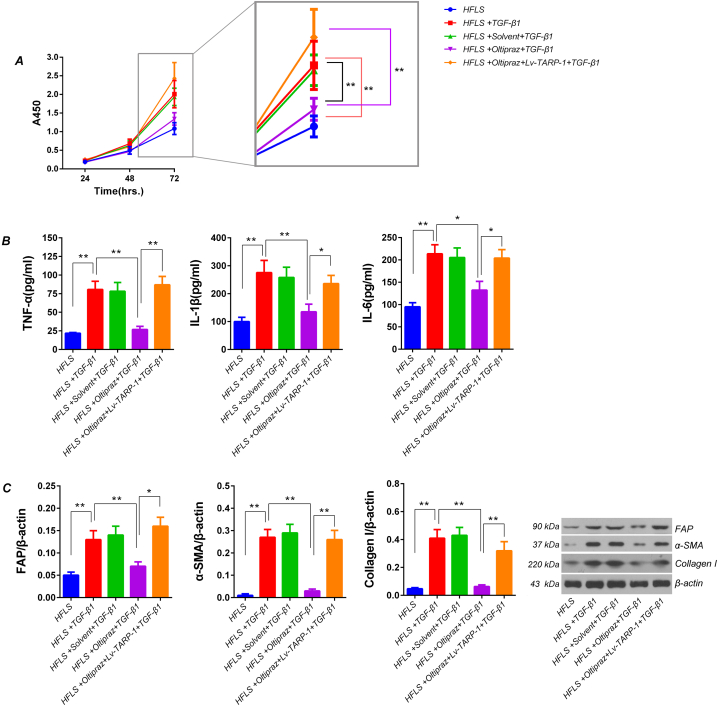
Detection of proliferation, inflammatory factor expression and secretion, and fibroblast activation proteins expression in HFLS with different treatments A)Proliferation was determined in HFLS using CCK-8 method. Due to the linear relationship between A450 value and cells proliferation, it can be directly used to represent cell proliferation activity. B)The contents of inflammatory factors in the supernatants of HFLS in each treatment group was detected by ELISA. C)Western blotting for proteins (FAP, α-SMA and Collagen I) expression in HFLS. **p < 0.01, *p < 0.05 (*t*-test). The tests were carried out in biological triplicates, and data are expressed as the mean ± SD.

The CCK-8 assay showed that TGF-β1 induction for 72h significantly increased the proliferation activity in HFLS (*p* < 0.01. *vs.* cell group, 72 h). The oltipraz pretreatment effectively inhibited the increased proliferation in HFLS induced by TGF-β1 (*p* < 0.01. *vs.* TGF-β1 induction group, 72 h), which cloud be significantly reversed by the Lv-TRAP-1 infection in HFLS (*p* < 0.01. *vs.* oltipraz pretreatment + TGF-β1 induction group, 72 h) (Fig. 4-A). The results of ELISA showed that TGF-β1 induction for 72 h significantly increased the contents of inflammatory cytokines TNF-α, IL-1β and IL-6 in the supernatants of HFLS (*p* < 0.01. *vs.* cell group). The oltipraz pretreatment could significantly inhibited the TGF-β1 caused increase of these inflammatory factors in the supernatants of HFLS (*p* < 0.01 vs. TGF-β1 induction group), and the effects of oltipraz could be significantly reversed by Lv-TRAP-1 infection (*p* < 0.01 vs. oltipraz pretreatment + TGF-β1 induction group) (Fig. 4-B). Western blotting results showed that TGF-β1 induction for 72 h significantly increased the proteins expression of FAP, α-SMA and Collagen I in HFLS (*p* < 0.01, *vs.* cell group), the oltipraz pretreatment could significantly inhibit the TGF-β1 caused increase of these proteins in HFLS (*p* < 0.01. *vs.* TGF-β1 induction group), and the effect of oltipraz were reversed by Lv-TRAP-1 infection (*p* < 0.01, *vs.* oltipraz pretreatment + TGF-β1 induction group. Fig. 4-C). These results collectively indicated that the Nrf2/miR338-3p/TRAP-1 pathway mediated the inhibitory effect of oltipraz on hyperactivation of HFLS.

## 4 Discussion

It is estimated that more than 240 million people worldwide suffer from OA. The pathological changes of the soft tissues around joints of OA patients usually lead to pain and stiffness and joint damage., which limits joint function and greatly reduces the quality of life in OA patients [8]. Studies have shown that 50 % of OA patients frequently have synovitis, the role of synovitis in OA pathogenesis should be taken seriously [9]. Synovial tissue is a type of specialized tissue that consists of a variety of cells, such as synovial fibroblasts, immune cells, vascular endothelial cells, etc. [10]. According to the current research, the incidence of OA is mostly related to genetics, age, trauma, inflammation, body mass index, joint use and other factors, but the study on the pathogenesis and treatment of OA is still in the exploratory stage, and its theoretical basis is relatively weak. Researchers have found that OA mainly was classified into the primary and secondary types, both of which are accompanied by varying degrees of subacute-chronic synovial hyperplasia and fibrosis. Under physiological and pathological conditions of OA, the excessive deposition of extracellular matrix (ECM) leads to increase of tissue hardness, excessive proliferation of synovial fibroblasts which resulting joint pain and stiffness [11].

The synovial fibroblasts are important part of synovium tissue system, plays an increasingly prominent role in OA.s. Studies have shown that FLS were the basic units to maintain the structure and function of the normal synovium [12]. Under the pathological conditions of OA, the high numbers of FLS in knee synovium can drive inflammation and are further activated. The activated FLS can promoted the progress of joint inflammation by secreting a large number of inflammatory factors and mediating the enrichment of leukocytes, which causing the hyperactivation FLS. Therefore, we can consider that the hyperactivation of FLS is not only a consequence of OA, but also a direct cause of amplifying local inflammatory reactions in joints and exacerbating the joint degeneration in OA patients [13,14].

Fibrosis can occur in multiple organs, with the main pathological changes being an increase in fibrous connective tissues and a decrease in parenchymal cells within the organ tissue, the continuous progression of fibrosis can lead to structural damage, functional decline, the failure of multiple organs, and even death. The inflammatory conditions in the joint cavity of OA patients can lead to the hyperactivation of FLS which causes synovial tissue hyperplasia and fibrosis [15]. These studies collectively demonstrate that inhibiting the hyperactivation of FLS is an effective means to suppress the pathological hyperplasia and fibrosis of synovial tissues in OA patients and improve their quality of life.

Nuclear factor E2 related factor 2 (Nrf2) plays an important role in maintaining the cell homeostasis and organ integrity. Nrf2 protein encoded by NFE2L2 gene is a transcription factor described to have a basic leucine zipper (bZIP) domain and the activated Nrf2 exhibits the phosphorylation and aggregation characteristics [16,17]. The bZIP is a highly conserved domain of 60–80 amino acids which can binds with MAF, Jun-D, C-Jun and other small molecular proteins in the nucleus to form heterodimers, and then binds to antioxidant response element (ARE) to activate the downstream target genes. Studies have shown that Nrf2 could slow down the process of liver fibrosis induced by TGF- β in vivo [18]. Palsamy et al. found that the resveratrol alleviated the renal injury in rats by activating Nrf2 and promoting the expression of its downstream genes [19]. It is reported that Avastin can play a key role in relieving cholestasis induced liver injury by activating Nrf2 [20]. The oleanolic acid derivatives has been proven to have the similar effects of Avastin [21,22]. In fact, the protective effect of activators targeting Nrf2 on fibrotic lesions of kidney and liver provides a new idea for new drug research and development for prevention and treatment of OA. TRAP-1 has been found to be associated with the TGF-β/SMAD signal pathway, and the most significant role of TRAP-1 is the ability to recruit SMAD4 to bind to SMAD2/3 to form complexes, which is phosphorylated and translocated into the nucleus where they activate the downstream effectors.

In our previous study, we found that the expression of TRAP-1 was significantly increased in FLS of OA patients compared to normals, and regulation of TRAP-1 at the post transcriptional level attributed to the decrease of miR338-3p. This is the first report of the involvement of miR338-3p/TRAP-1 axis in the regulation of Synovial Fibroblast activation in patients with OA [9]. Although the study showed that miR338-3p has the potential to become a biological target for OA treatment, the causes of decrease in expression level of miR338-3p in FLS of OA patients still need to be further explored.

In this study, we showed that the decrease of miR338-3p in HFLS of OA patients was attributed to the decreased activity of its upstream regulator Nrf2. Based on the research data, we logically and firstly proved that Nrf2/miR338-3p/TRAP-1 pathway participated in hyperactivation of HFLS of patients with OA, that is a great progress in the study of regulation mechanism of OA and we might use TRAP-1 as the target for the development of new drugs and therapy of OA in future. In fact, we did effectively inhibit the activation of HFLS induced by TGF-β1 by using the agonist of Nrf2, oltipraz. Specifically, oltipraz significantly blocked the up-regulation of expression of fibroblast proteins (FAP, α-SMA and Collagen I) and release of inflammatory factors (TNF-α, IL-1β and IL-6), and enhancement of proliferative activity in HFLS induced by TGF-β1. Importantly, the effect of oltipraz on activation of HFLS was significantly reversed by overexpression of exogenous TRAP-1 in HFLS, indicating that the Nrf2/miR338-3p/TRAP-1 pathway is significant for suppression of hyperactivation of HFLS, and the pathway members have potential to be used as targets for new drugs development of OA.

Up to now, the clinical therapeutic drugs of OA mainly include non-steroidal anti-inflammatory drugs (NSAIDs), opioid analgesics, compound analgesics, glucocorticoids, sodium hyaluronate, and medical chitosan, etc. Although these drugs may have a positive effect on alleviating joint soft tissue fibrosis in OA patients, they mostly aim to alleviate pain. As far as I know, there are no new drug research and development plans that focus on soft tissue fibrosis. In fact, the cartilage and synovial fibrosis caused by chronic inflammation and tissue damage have only recently been emphasized in OA. Synovial tissues of OA often undergo harden and thicken, but the mechanism of fibrosis is still unclear. Synovial fibroblasts transform into myofibroblasts and excessive secrete the extracellular matrix (ECM). TGF-β1 is one of the main participants in fibrosis and plays a crucial role in the activated fibrosis cascade in OA.

Recent studies have collectively shown that Nrf2 can participate in the regulation of fibrosis progression in lung and liver tissues by diverse ways, and Nrf2 exhibits a positive effect in inhibiting tissue fibrosis [[23], [24], [25]]. Our study is the first to investigate the mechanism of Nrf2 involvement in synovial tissue fibrosis in OA patients. Oltipraz, as an NRF2 agonist, has been approved by the Food and Drug Administration (FDA) for phase III clinical trials in the treatment of fatty liver and liver fibrosis. Researchers have found that this small molecule can have a positive improvement effect on liver fibrosis in patients with fatty liver disease [26]. Another independent study published in Biomaterials showed that PLGA nanoparticles loaded with oltipraz could effectively improve the acute renal injury and fibrosis [27]. However, as of now, there have been no reports on the application of oltipraz in the treatment of OA. Nevertheless, our research still focuses on elucidating the new mechanism of NRF2/miR-338-3p/TARP-1 pathway involvement in joint soft tissue lesions in OA patients. Targeting NRF2, it is evident that the search for effective drugs against synovial fibrosis in OA patients should not be limited to oltipraz, and other types of Nrf2 agonists should also be paid attention to in future research.

## 5 Conclusions

In summary, we confirmed for the first time that Nrf2/miR338-3p/TRAP-1 pathway was involved in the hyperactivation of HFLS under OA pathological conditions. The significance of this study is mainly embodied in the theory of Nrf2 being used as target for therapy and new drug developments of OA from the perspective of combating pathological hyperplasia and fibrosis of joint soft tissues. Furthermore, the experience and ideas in the study have great clinical reference value, and they suggested that the potential role of Nrf2 protein agonists such as oltipraz in treatments of OA are worthy of investigate and research.

## Data availability statement

Data will be made available on request.

## Funding statement

The project was financially supported by the 2020 Jiangsu Province's fifth phase "333 project" training fund support project (No. BRA2020215).

## CRediT authorship contribution statement

**Peng Jie:** Data curation, Investigation, Methodology, Writing – original draft, Writing – review & editing. **Ya Wu:** Data curation, Software, Writing – original draft. **Changzhi Song:** Conceptualization, Funding acquisition, Methodology, Project administration, Validation. **Yi Cheng:** Data curation, Formal analysis, Investigation. **Yunfei Liu:** Data curation, Formal analysis, Investigation, Software. **Kang Chen:** Data curation, Formal analysis, Investigation, Validation.

## Declaration of competing interest

The authors declare the following financial interests/personal relationships which may be considered as potential competing interests.
