# Supplementary material for: Mechanism of Nrf2/miR338-3p/TRAP-1 pathway involved in hyperactivation of synovial fibroblasts in patients with osteoarthritis
Source: Heliyon. 2023 Oct 21;9(11):e21412. doi: 10.1016/j.heliyon.2023.e21412 (PMC10618819; doi:10.1016/j.heliyon.2023.e21412)
Supplement: Multimedia component 1 [file mmc1.pptx]

## Slide 1
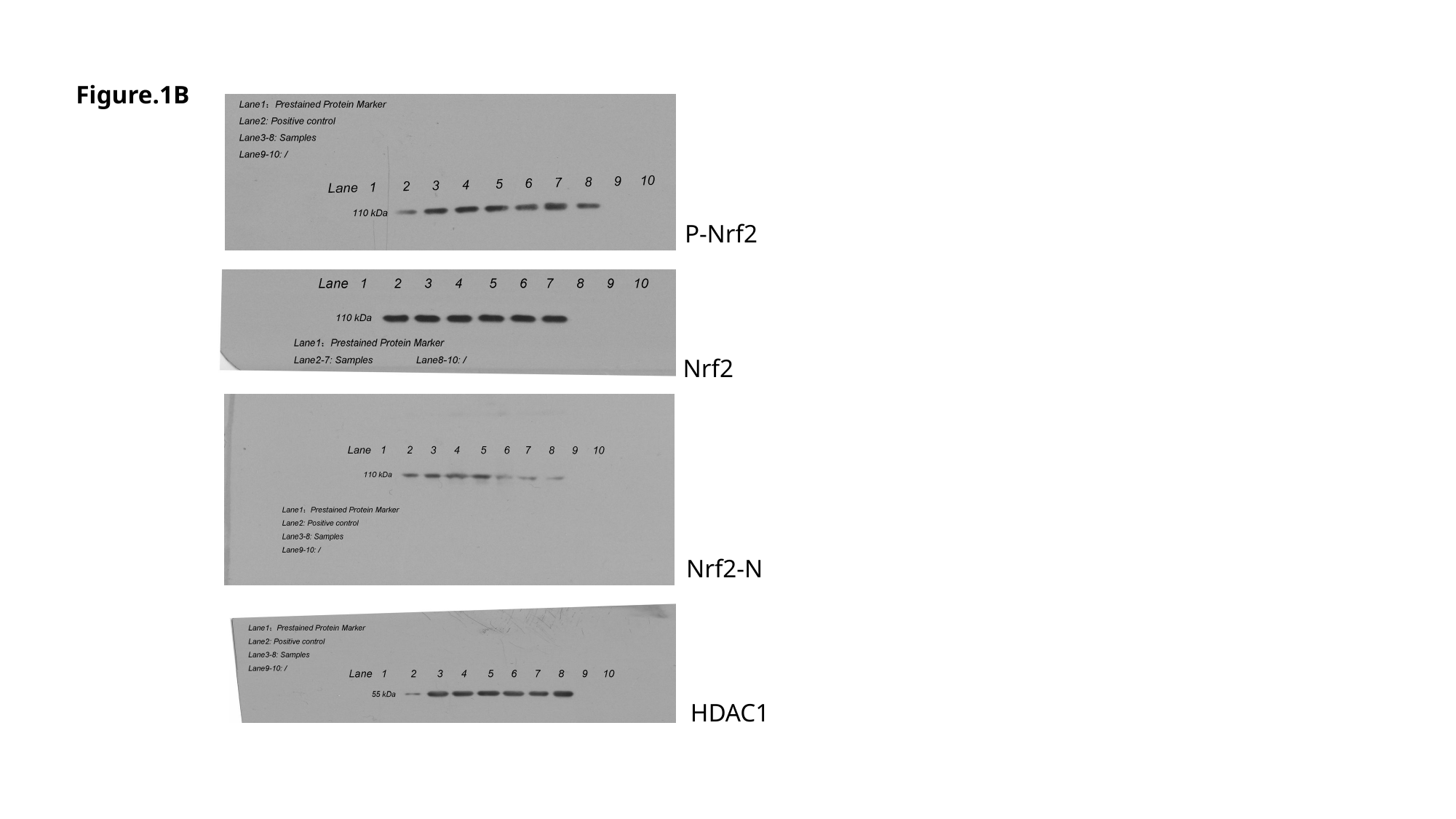

Figure.1B
P-Nrf2
Nrf2
Nrf2-N
HDAC1

## Slide 2
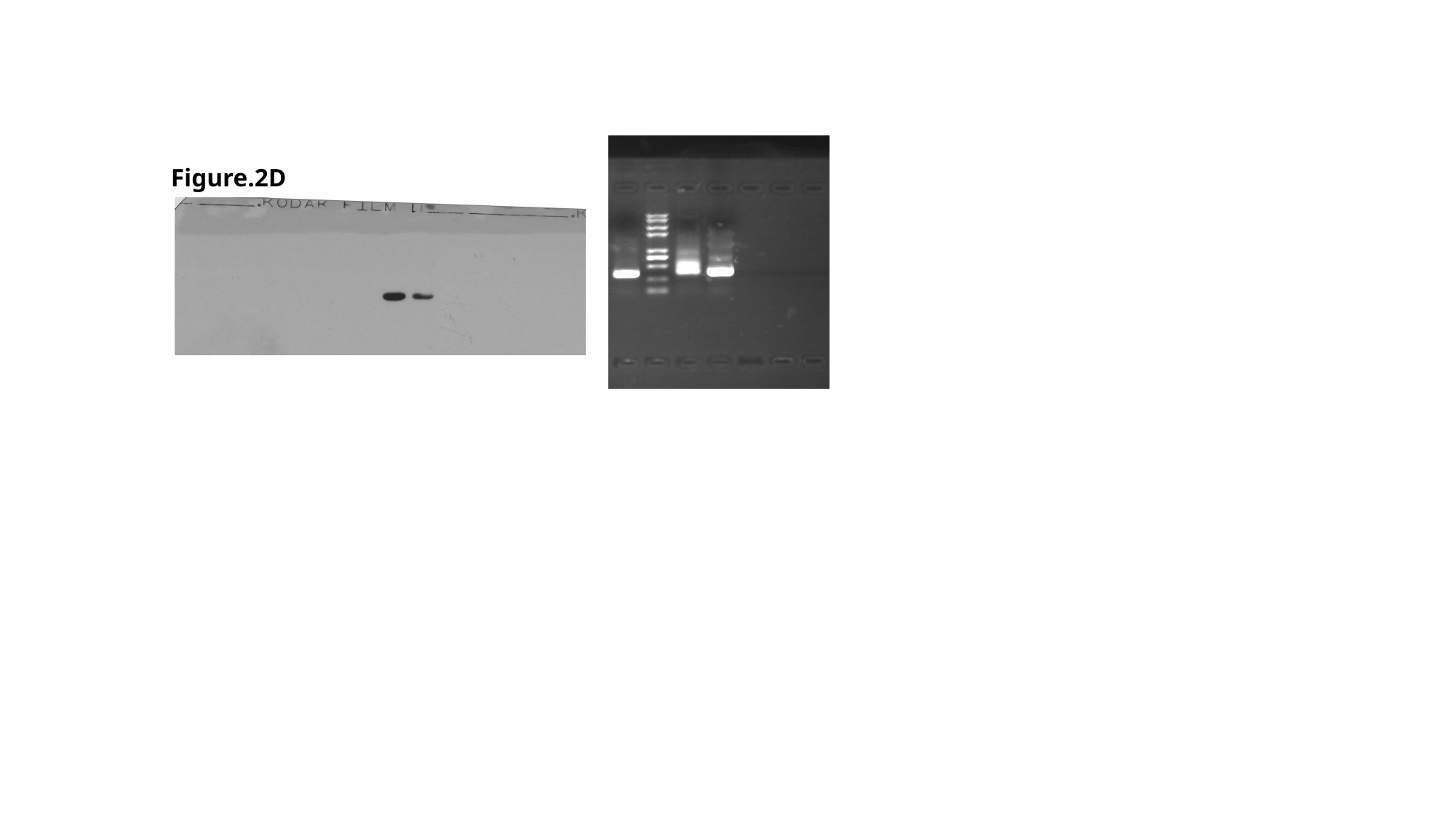

Figure.2D

## Slide 3
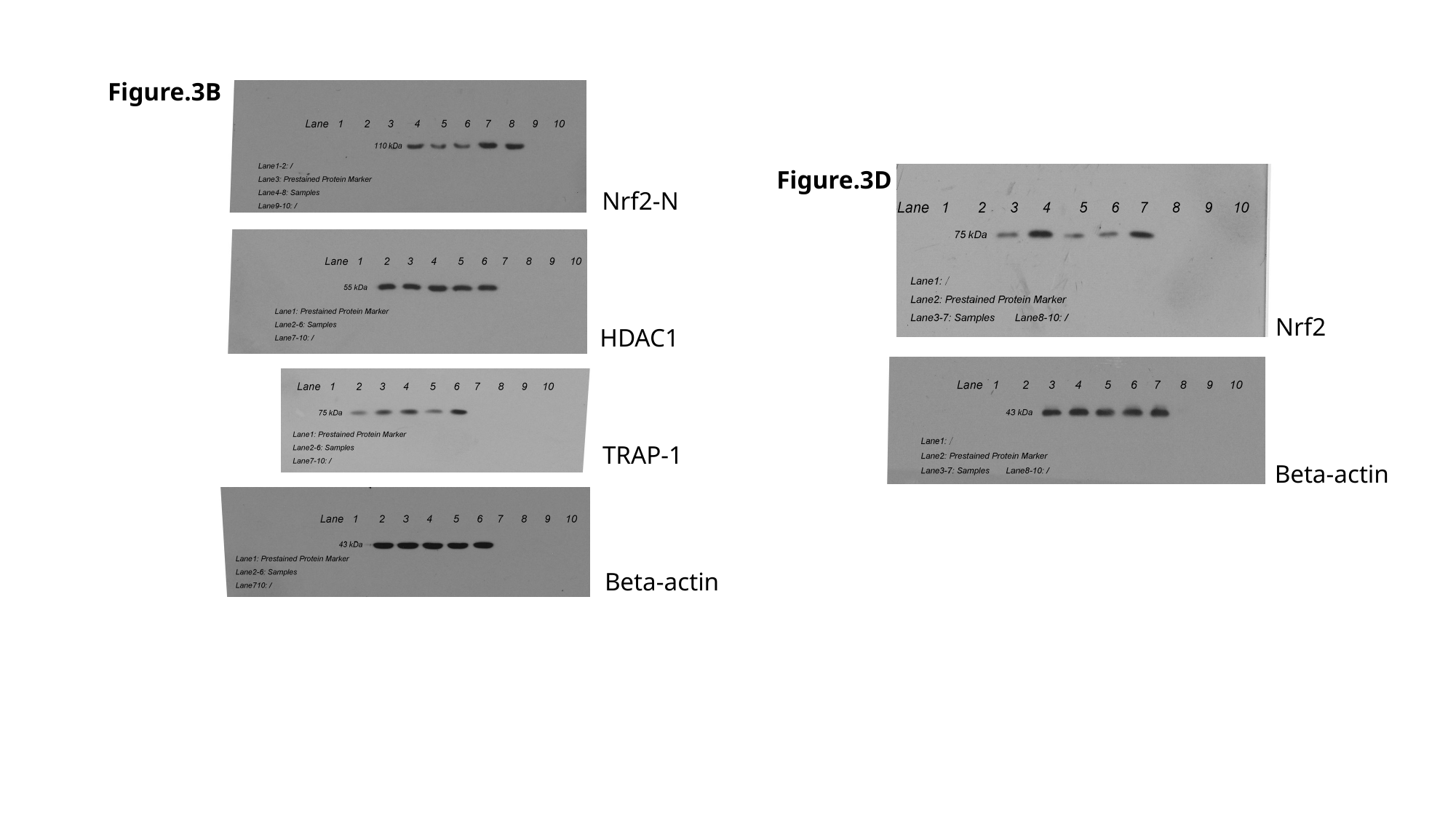

Figure.3B
Figure.3D
Nrf2-N
Nrf2
HDAC1
TRAP-1
Beta-actin
Beta-actin

## Slide 4
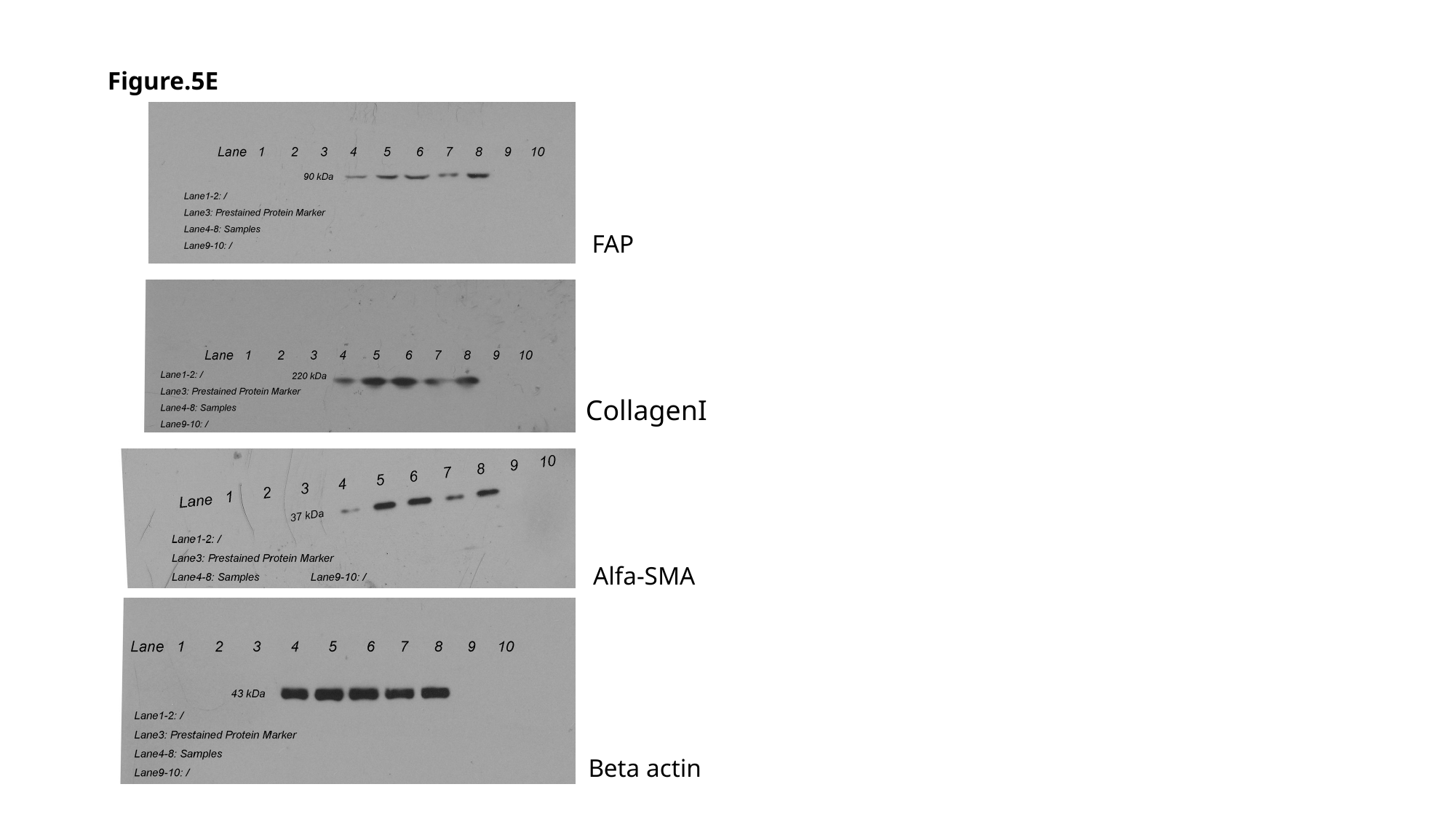

Figure.5E
FAP
CollagenI
Alfa-SMA
Beta actin
